# Air Pollution and Atherosclerosis: New Evidence to Support Air Quality Policies

**DOI:** 10.1371/journal.pmed.1001432

**Published:** 2013-04-23

**Authors:** Nino Künzli

**Affiliations:** 1 Swiss Tropical and Public Health Institute (Swiss TPH), Basel, Switzerland; 2 University of Basel, Basel, Switzerland

## Abstract

Nino Künzli discusses a large new cohort study that provides some of the first human evidence for the impact of air pollution on the development of atherosclerosis.

**Linked Research Article**

This Perspective discusses the following new study published in *PLOS Medicine*:

Adar S, Sheppard L, Vedal S, Polak JF, Sampson PD, et al. (2013) Fine Particulate Air Pollution and the Progression of Carotid Intima-medial Thickness: A Prospective Cohort Study from the Multi-Ethnic Study of Atherosclerosis and Air Pollution. PLoS Med 10(4): e1001430. doi:10.1371/journal.pmed.1001430

In a prospective cohort study, Sara Adar and colleagues find that decreasing levels of fine particulate matter in multiple US urban areas are associated with slowed progression of intima-medial thickness, a surrogate measure of atherosclerosis.

Increasing attention is being paid to the subject of air pollution, with the bulk of previous work being done in animal models suggesting that exposure to air pollution causes atherosclerosis—stiffening and calcification of the arteries—in rabbits and mice [1],[2]. The new findings of Sara Adar and colleagues, published in this week's *PLOS Medicine* [3], take us a step forward in clarifying the broader implications of air pollution by offering further evidence in humans that ambient particulate matter (PM) contributes to the development of cardiovascular disease (CVD).

CVDs are now the world's leading causes of death [4]. The most important underlying pathology resulting in CVD is atherosclerosis, known to be a life-long process with a long and silent pre-clinical phase. It has been known since the last century that ambient air pollution can trigger acute cardiovascular morbidities [5], and a comparative risk assessment of established triggers of myocardial infarctions concluded that a rather substantial fraction of these acute and life-threatening events can be attributed to current levels of air pollution [6]. However, it is of importance to understand the causes of atherosclerosis, given that its prevention or deceleration could drastically delay and reduce the burden of CVDs.

The hypothesis that ambient air pollution could play a role in these chronic processes was first addressed and confirmed in rabbits and mice [1],[2]. Adar et al. have now provided important human-based results based upon the Multi-Ethnic Study of Atherosclerosis and Air Pollution (MESA). It is only the second longitudinal study about the impact of air pollution on the progression of atherosclerosis and is the first one based on a general population sample free of CVD at study onset. The authors here share their early findings of MESA, which examined associations of common carotid artery intima-medial thickness (IMT), a surrogate of atherosclerosis, with long-term PM2.5 concentrations among over 5,300 people over an average of two-and-a-half years. They report that higher long-term PM2.5 concentrations are associated with increased IMT progression and that greater reductions in PM2.5 are related to slower IMT progression. MESA replicates findings observed in the first longitudinal study, which was based on data from five clinical trials conducted in volunteers from Los Angeles [7]. Both research teams used IMT as a marker of atherosclerosis [8]. With only two longitudinal studies, it is worth discussing three important issues that need to be addressed in the future, namely the contributing role of noise, the link between cross-sectional and longitudinal findings, and the identification of susceptibility factors.

## Future Research Needs

First is the issue of noise. Although the MESA findings are said not to be sensitive to adjustment for perceived noise, the discussion about the atherogenic role of traffic-related night-time noise (e.g., via its impact on blood pressure) and its potential confounding effect in cardiovascular air pollution research is not settled and must be explored in future research. MESA used “perceived noise," which is attractive if the subjective experience of noise matters more than the objective levels. Other studies use instead objective measures of noise; however, they are estimated outdoors at the façade. Both approaches fail to capture personal noise exposure during night time, which heavily depends on coping behaviours such as sleeping away from the noise, keeping windows closed, wearing ear plugs, or taking sleeping pills—adaptations that may indeed be determined by the perception of noise. Given that the correlation between personal night-time noise and air pollution exposure is not known, claims that air pollution findings are not confounded by noise remain at least uncertain.

Second is the issue of consistency between cross-sectional and longitudinal findings. These two longitudinal studies now provide the first data to connect and interpret cross-sectional results [7],[9]–[11]. The use of IMT as a marker of the cumulated long-term exposures to atherogenic risk factors is appealing [8]. Depending on those risk patterns, the IMT of a participant may progress slower or faster, and Figure 1 of Adar and colleagues' paper provides the respective data [3]. Are the cross-sectional associations with PM2.5 consistent with the observed impact on progression? Adar and colleagues include two approaches (seen in their Tables 2 and 3), and for each an “overall" estimate and a “within-city" estimate for cross-sectional and progression data are provided. The Los Angeles studies provide another pair of data [7],[11]. One can use those five pairs of data to calculate the number of years needed (progression) to reach the observed cross-sectional contrasts, given e.g., long-term exposure to 10 µg/m^3^ higher PM2.5 levels. For the within-city main model, the derived time is extremely short (1 month). Based on data from the Los Angeles trials, one would need 12 years to reach the published cross-sectional differences. The MESA “overall" models result in 15 to 22 years—thus, we end up with a broad range that remains hard to explain but should be further explored.

It is also a challenge to connect findings given the very substantial differences in the absolute progression of IMT reported in these different study populations. In MESA, annual IMT progression was some 5–10 times larger than in the Los Angeles trials (10–20 µm/y compared to 2 µm/y) despite similar IMT means, follow-up duration, and age structures. This may relate to lab-specific differences in sonographic IMT measurement methods and it should not affect the validity of the air pollution analyses within studies. But the issue also needs careful evaluation and comparison with other studies prior to translating the IMT results into health impact assessments.

Third is the issue of susceptibility factors. While women are better protected against atherogenesis during the fertile life span, the pathology becomes relevant and is a leading cause of female morbidity and death in peri- and post-menopausal life. Like in the Los Angeles clinical trial study [7], air pollution effects in MESA were larger in women. This was also observed in the cross-sectional analyses of the Los Angeles trials [11], with associations being particularly strong in women >60 years of age. However, other cross-sectional studies could not replicate this pattern [8]. Future research should focus in more general terms on the identification of modifying factors. A better understanding of the role of metabolic pathologies such as diabetes and obesity is particularly warranted given the epidemic of these conditions [12]. The Los Angeles study observed the largest effects in the trial conducted with diabetics. The MESA study reported somewhat larger estimates among diabetics too, but no modification by obesity. If obese people are more affected by air pollution, the ongoing obesity epidemic could jeopardize the benefits of air quality improvements now seen in many countries [12].

In sum, the MESA study further supports an old request to policy makers, namely that clean air standards ought to comply at least with the science-based levels proposed by the World Health Organization [13]. And we know it works: better air quality improves health [14]—in rabbit, mice, men, and women alike.

## Abbreviations

CVD: cardiovascular disease
IMT: intima-medial thickness
MESA: Multi-Ethnic Study of Atherosclerosis and Air Pollution
PM: particulate matter
